# MicroRNA-132 Negatively Regulates Palmitate-Induced NLRP3 Inflammasome Activation through FOXO3 Down-Regulation in THP-1 Cells

**DOI:** 10.3390/nu9121370

**Published:** 2017-12-18

**Authors:** Hye-Eun Byeon, Ja Young Jeon, Hae Jin Kim, Dae Jung Kim, Kwan-Woo Lee, Yup Kang, Seung Jin Han

**Affiliations:** 1 Institute of Medical Science, Ajou University School of Medicine, 164 World cup-ro, Yeongtong-gu, Suwon 16499, Korea; 110236@aumc.ac.kr; 2 Department of Endocrinology and Metabolism, Ajou University School of Medicine, 164 World cup-ro, Yeongtong-gu, Suwon 16499, Korea; twinstwins@hanmail.net (J.Y.J.); jinkim@ajou.ac.kr (H.J.K.); djkim@ajou.ac.kr (D.J.K.); LKW65@ajou.ac.kr (K.-W.L); 3 Department of Physiology, Ajou University School of Medicine, 164 World cup-ro, Yeongtong-gu, Suwon 16499, Korea; kangy@ajou.ac.kr

**Keywords:** palmitate, inflammasome, microRNA, miR-132

## Abstract

Saturated fatty acids were proposed to activate the NLRP3 inflammasome, a molecular platform that mediates the processing of interleukin (IL)-1β and IL-18. However, the mechanisms underlying the miRNA-mediated regulation of palmitate (PA)-induced inflammasome activation are unclear. We examined the role of miR-132 in PA-induced NLRP3 inflammasome activation in THP-1 cells. To understand the regulatory role of miR-132 in inflammasome activation, we either overexpressed or suppressed miR-132 in THP-1 cells that expressed the NLRP3 inflammasome in response to stimulation by PA. We analyzed the mRNA and protein levels of NLRP3, caspase-1 p10, IL-18, and IL-1β; caspase-1 activity; and IL-1β secretion. The presence of PA activated the NLRP3 inflammasome and increased miR-132 expression. Overexpression of miR-132 reduced caspase-1 p10, IL-18, and IL-1β, while the suppression of miR-132 enhanced inflammasome activation. In addition, miR-132 regulated the mRNA and protein expression of FOXO3, which is a potential target of miR-132 in these cells. FOXO3 suppression by small interfering RNA decreased NLRP3 inflammasome activity stimulated by PA. Knockdown of FOXO3 attenuated NLRP3 inflammasome activation by the miR-132 inhibitor. Based on these findings, we conclude that miR-132 negatively regulates PA-induced NLRP3 inflammasome activation through FOXO3 down-regulation in THP-1 cells.

## 1. Introduction

Obesity and its comorbidities, including type 2 diabetes and cardiovascular disease, are associated with a state of chronic low-grade inflammation, characterized by up-regulated cytokine production and inflammation [1]. Inflammation is an innate immune response to repair tissue injury, and is predominantly mediated via myeloid cells such as monocytes, macrophages, and neutrophils. Inflammation is triggered by pathogen-associated molecular patterns and endogenous substances that are released during tissue or cell damage (danger-associated molecular patterns) [2,3]. The inflammasome is an important part of our innate immune system that responds to danger signals that are sensed by many different NOD-like receptors (NLRs) [4]. Inflammasomes are molecular platforms that modulate innate immune functions by activating caspase-1 and by catalyzing the proteolytic processing and secretion of interleukin (IL)-1β and IL-18 [5]. To date, nucleotide-binding domain leucine-rich repeat containing family, pyrin domain-containing 3 (NLRP3) is the most extensively studied inflammasome and member of the NLR family, which consists of 22 members and three distinctive subfamilies [5]. 

The excessive intake of saturated fatty acids (SFAs) is an important factor that contributes to metabolic disorders. In addition, SFA overload induces stress signaling pathways such as endoplasmic reticulum stress, reactive oxygen species production, and apoptosis, as well as inflammatory pathways [6]. During inflammation, SFAs promote chronic inflammatory responses by engaging toll-like receptors (TLRs) and inducing the nuclear factor-kappaB (NF-κB)-dependent production of inflammatory cytokines such as tumor necrosis factor-α [7,8]. Furthermore, SFAs were recently identified as triggers of the NLRP3 inflammasome in macrophages; additionally, they increase the secretion of IL-1β and IL-18, both of which play a role in the development of obesity-induced insulin resistance [9,10]. However, the precise mechanism of SFA-induced NLRP3 inflammasome activation is not fully understood. 

Recently, microRNA (miRNA)-mediated posttranscriptional regulation was shown to play an important role in controlling gene expression and transcription. miRNAs are endogenous, small, noncoding RNAs approximately 18–25 nucleotides in length that post-transcriptionally regulate gene expression. miRNAs act by forming imperfect base pairs with sequences in the 3′-untranslated regions of protein-coding mRNAs to prevent protein synthesis by repressing translation or inducing mRNA degradation [11]. miRNAs are associated with diverse biological processes, ranging from development to differentiation and regulation of the immune system [12]. The role of miRNAs in inflammation regulation has primarily been studied for TLR signal transduction pathways. Several miRNAs are involved in TLR activation and target mRNAs encoding components of the TLR-signaling system itself [13]. Among these miRNAs, miR-132 was found to be markedly induced by lipopolysaccharide (LPS) treatment in THP-1 cells; it plays a role in suppression of the inflammatory response [14]. In addition, miR-132 has been shown to regulate antiviral innate immunity by targeting p300 [15]. However, studies showing the participation of miR-132 in SFA-induced inflammasome activation are lacking. Therefore, this study aimed to elucidate the potential involvement of miR-132 in palmitate (PA)-induced NLRP3 inflammasome activation in THP-1 cells. 

## 2. Materials and Methods 

### 2.1. Mice and Reagents 

Male C57BL/6 mice (5 weeks old) were purchased from Orient (Anyang, Korea) and acclimated under controlled conditions for 1 week before the experiment. The animals were housed in cages located in temperature-controlled rooms under 12:12 h light–dark cycle conditions, and fed water and food ad libitum. All animal procedures were approved by the Ajou University Animal Care Ethics Committee (Ethics No. 2017-0059; 24 October 2017).

PA and LPS were purchased from Sigma-Aldrich (St. Louis, MO, USA). PA–bovine serum albumin (BSA) conjugates were prepared as described previously [16]. Briefly, PA–BSA conjugates were generated by soaping PA with sodium hydroxide (NaOH) and mixing with BSA. A 20 mM solution of palmitate in 0.01 M NaOH was incubated at 70 °C for 30 min, and the fatty acid soaps were then complexed with 5% fatty acid-free BSA in phosphate-buffered saline in a 1:3 volume ratio. The complexed fatty acids consisted of 5 mM PA and 3.75% BSA, but the molar ratio of PA to BSA was 8:1. The PA–BSA conjugates were diluted in 10% fetal bovine serum (FBS)-containing medium and administered to cultured cells at 100–400 μM PA.

### 2.2. Cell Culture, Isolation of Peritoneal Macrophages, and PA Treatment

THP-1 cells were cultured in Roswell Park Memorial Institute (RPMI) 1640 medium (2 g/L of glucose) supplemented with 10% FBS, 100 U/mL of penicillin, and 100 μg/mL of streptomycin (Life Technologies, Inc., Gaithersburg, MD, USA). The cells were seeded in 6-well plates at a density of 4 × 10^5^ cells per well and incubated for 2–24 h in cell culture media containing different concentrations of PA. Control cells were incubated with the same medium containing the same amount of solvent (BSA) used to dissolve the PA.

Peritoneal exudate cells obtained from C57BL/6 male mice were washed twice and resuspended in the same medium as above. Macrophages were isolated from the peritoneal exudate cells as described previously [17]. The peritoneal exudate cells were seeded on Teflon-coated petri dishes (100 × 15 mm) at densities of 5–6 × 10^5^ cells/cm^2^, and the macrophages were allowed to adhere for 2–3 h at 37 °C in a 5% CO_2_ humidified atmosphere. The non-adherent cells were removed by washing the dishes twice with 10 mL prewarmed medium and incubating the dishes for 10 min at 4 °C. The supernatants were carefully removed and discarded, and the plates were washed once with prewarmed Dulbecco’s phosphate-buffered saline solution (PBS) (Gibco Laboratories, Gaithersburg, MD, USA). Cold PBS (15 mL) containing 1.5% FBS was added, followed by the addition of 0.3 mL 0.1 M EDTA (pH 7.0). The viability of the detached cells was assessed by trypan blue exclusion, and the proportion of macrophages was determined after cytoplasmic staining with acridine orange using fluorescence microscopy. More than 95% of the cells were viable, and the cell preparations contained >95% macrophages.

### 2.3. Quantification of miRNA and mRNA Expression 

For the quantitative analysis of miRNA expression, quantitative RT-PCR was performed using a TaqMan MicroRNA Reverse Transcription Kit, the TaqMan MicroRNA Assay (primer/probe sets for miRNAs), and TaqMan Universal PCR Master Mix (Applied Biosystems, Foster City, CA, USA), according to the manufacturer’s protocols. Relative miRNA expression was normalized to U6 small nuclear RNA. The results were analyzed using the 2^−∆∆ CT^ method. 

For mRNA analysis, cDNA was prepared using a PrimeScriptTM 1st Strand cDNA Synthesis Kit and amplified using a Takara SYBR Green I Kit (Takara Bio Inc., Otsu, Japan) according to the manufacturer’s instructions. PCR was conducted using the ABI PRISM 7900 HT sequence detection system and ABI PRISM SDS 2.0 software (Applied Biosystems). GAPDH expression levels were used as an internal control. Expression levels were calculated using the 2^−∆∆ CT^ method. The following primers were used (Table 1).

### 2.4. Transfection with miRNA Inhibitor and Mimic

THP-1 cells were cultured in a 6-well culture plate at a density of 4 × 10^5^ cells per well for 24 h. The cells were then transfected with 20 nM mimic negative control, miR-132 mimics, an inhibitor negative control, or an miR-132 inhibitor (Ambion^™^; Life Technologies, Carlsbad, CA, USA) using Lipofectamine RNAiMAX (Thermo Fisher Scientific, Waltham, MA, USA), according to the manufacturer’s instructions for 48 h.

### 2.5. FOXO3 and NF-κB Small Interfering RNA (siRNA) Transfection

The siRNA sequences were as follows: human FOXO3 siRNA I, 5′-ACUCCGGGUCCAGCUCCAC-3′ (sense) and 5′-GUGGAGCUGGACCCGGAGU-3′ (antisense); human FOXO3 siRNA II, 5′-UAGAAUUGGUGCGUGAACGGAAGUC-3’ (sense) and 5′-GACUUCCGUUCACGCACCAAUUCUA-3′ (antisense); and mouse FOXO3 siRNA, 5′-UGAUGAUCCACCAAGAGCUCUUGCC-3′ (sense) and 5′-GGCAAGAGCUCUUGGUGGAUCAUCA-3′ (antisense). siRNA duplexes were chemically synthesized (Bioneer, Daejeon, Korea). Human NF-κB siRNA was purchased from Cell Signaling (catalog no. 6261; Danvers, MA, USA). THP-1 cells or primary murine macrophages were transiently transfected with FOXO3 siRNA/NF-κB siRNA and a control siRNA for 48 h at a final concentration of 20 nM using Lipofectamine RNAiMAX (Thermo Fisher Scientific, Agawam, MA, USA) according to the manufacturer’s instructions.

### 2.6. Preparation of Whole, Nuclear, and Cytosolic Extracts and Supernatants, and Immunoblotting

Immunoblotting was performed using cell lysates and cell supernatants. Cellular proteins were extracted from whole cell lysates using radioimmunoprecipitation buffer (50 mM Tris-HCl at pH 7.5, 150 mM NaCl, 1% NP-40, 0.5% deoxycholate, 0.1% sodium dodecyl sulfate (SDS), and protease inhibitor cocktail). Nuclear and cytosolic proteins were extracted using a Nuclear/Cytosolic Fraction kit according to the manufacturer’s instructions (catalog no. AKR-171; Cell Biolabs, San Diego, CA, USA). Protein concentrations in the cell lysates were determined using protein assay kits (Bio-Rad, Hercules, CA, USA). An equal volume of 2 × SDS sample buffer (125 mM Tris-HCl, (pH 6.8), 4% SDS, 4% 2-mercaptoethanol, and 20% glycerol) was added to the cell lysates. Equivalent amounts of protein (10–30 μg) were loaded onto 10–15% polyacrylamide gels, fractionated by electrophoresis, and transferred onto polyvinylidene fluoride membranes (Millipore, Bedford, MA, USA). Membranes were sequentially incubated with primary and secondary antibodies in Tris-buffered saline containing 0.05% Tween-20 supplemented with 5% (*w*/*v*) non-fat dry milk. Immunoreactive bands were developed using an enhanced chemiluminescence detection system (Amersham Pharmacia Biotech, Arlington Heights, IL, USA). Band intensities were determined by densitometric analysis using ImageJ (NIH, Bethesda, MD, USA). 

To measure secreted caspase-1 p10, IL-18, and IL-1β in cell culture supernatants, supernatants were concentrated, precipitated using trichloroacetate, and analyzed by immunoblotting. The primary antibodies used were as follows: anti-NLRP3 (catalog no. AG-20B-0014; Adipogen, Incheon, Korea), anti-caspase-1 p10 (catalog no. sc-56036 for human, sc-514 for mouse; Santa Cruz Biotech, Santa Cruz, CA, USA), human anti-IL-1β (catalog no. MAB201; R&D Systems, Minneapolis, MN, USA), mouse anti-IL-1β (catalog no. ab9722; Abcam, Cambridge, MA, USA), anti-IL-18 (catalog no. ab68435; Abcam), anti-Foxo3a (catalog no. 2497; Cell Signaling), anti-nuclear factor (NF)-kB p65 (catalog no. sc-8008; Santa Cruz Biotech), anti-histone H1 (catalog no. sc-8030; Santa Cruz Biotech), and anti-β-actin (catalog no. sc-81178; Santa Cruz Biotech).

### 2.7. Enzyme-Linked Immunosorbent Assay (ELISA)

The amounts of IL-1β and caspase-1 in the culture supernatants were determined by quantitative ELISA kits according to the manufacturer’s instructions (human IL-1β, BioLegend Inc., San Diego, CA, USA; and caspase-1, R&D Systems). Mouse caspase-1 and IL-1β activity were measured using anti-caspase-1 p10 (sc-514; Santa Cruz Biotechnology) and anti-IL-1β (ab9722; Abcam).

### 2.8. Statistical Analyses

All data are presented as the means ± standard error of the mean (SEM) of three independent experiments. For comparisons between two groups, the Student’s *t*-test was used. Multi-group comparisons of mean values were analyzed by a one-way analysis of variance followed by LSD post hoc test. Statistically significant differences were defined as *p* < 0.05. 

## 3. Results

### 3.1. PA Induces NLRP3 Inflammasome Activation in THP-1 Cells and Primary Murine Macrophages

**Figure 1:**
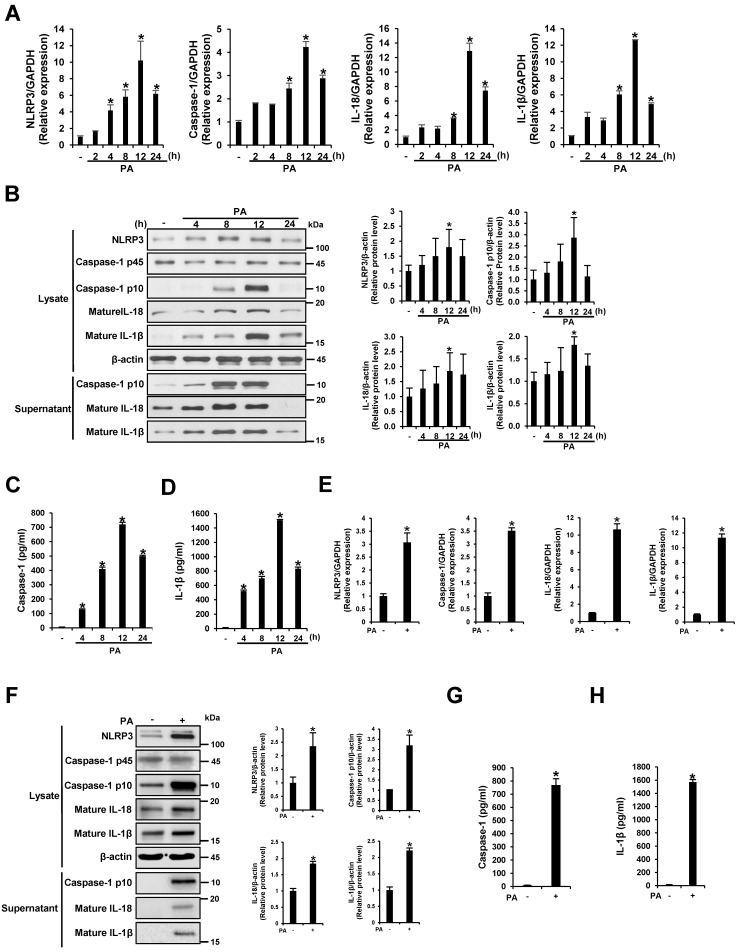
Palmitate (PA) induces NLRP3 inflammasome activation. (**A**) THP-1 cells were treated with 200 μM PA for 2, 4, 8, 12, and 24 h. The mRNA levels of NLRP3, caspase-1, interleukin (IL)-18, and IL-1β were examined by quantitative RT-PCR; (**B**) THP-1 cells were treated with 200 μM PA for 4, 8, 12, and 24 h. Cell lysates and supernatants were subjected to immunoblotting using antibodies specific for NLRP3, caspase-1, IL-18, IL-1β, and β-actin. Relative protein levels were analyzed with ImageJ; (**C**,**D**) The levels of caspase-1 and IL-1β in the culture supernatants were determined by ELISA; (**E**–**H**) Primary murine macrophages were treated with 200 μM PA for 12 h; (**E**) The mRNA levels of NLRP3, caspase-1, IL-18, and IL-1β were measured by quantitative RT-PCR; (**F**) Cell lysates and supernatants were subjected to immunoblotting using antibodies specific for NLRP3, caspase-1, IL-18, IL-1β, and β-actin. Relative protein levels were analyzed with ImageJ; (**G**, **H**) The levels of caspase-1 and IL-1β in the culture supernatants were determined by ELISA. * *p* < 0.05 compared with PA-untreated cells.

To investigate the effect of PA on the transcription of NLRP3 inflammasome components, THP-1 cells were treated with 200 μM of PA–BSA conjugate for 2, 4, 8, 12, and 24 h. As shown in Figure 1A, the mRNA levels of NLRP3, caspase-1, IL-18, and IL-1β increased gradually in a time-dependent manner for 12 h at all concentrations. Western blotting was then performed to analyze the protein levels of these genes in the cell lysates and culture supernatants after THP-1 cells were treated with 200 μM PA for 4, 8, 12, and 24 h. The protein expression of the p10 fragment of mature caspase-1, IL-18, and IL-1β was enhanced in a similar manner as mRNA expression (Figure 1B). Furthermore, significant levels of the p10 fragment of mature caspase-1, IL-18, and IL-1β were released into the culture supernatant (Figure 1B). ELISA analysis revealed that PA enhanced caspase-1 activity and IL-1β secretion (Figure 1C,D). In addition, we confirmed that PA induced NLRP3 inflammasome activation in primary murine macrophages, which resembled SFA-overloaded macrophages (Figure 1E–H). These results suggest that PA induced NLRP3 inflammasome activation, which induced NLRP3 expression, caspase-1 activation, and caspase-1-dependent production of IL-18 and IL-1β in THP-1 cells and primary murine macrophages.

### 3.2. PA Up-Regulates miR-132 Expression in THP-1 Cells and Primary Murine Macrophages

**Figure 2:**
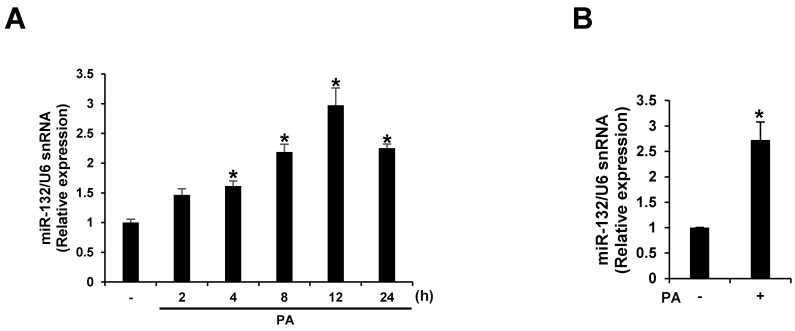
PA up-regulates miR-132 expression. (**A**) THP-1 cells were treated with 200 μM PA for 2, 4, 8, 12, and 24 h; (**B**) Primary murine macrophages were treated with 200 μM PA for 12 h. miR-132 expression was analyzed by quantitative RT-PCR. * *p* < 0.05 compared with PA-untreated cells.

We screened miRNAs up-regulated by PA treatment using microarray analyses. miR-132 expression was up-regulated >10-fold following PA treatment in THP-1 cells. We confirmed the effect of PA treatment on the expression of miR-132 using quantitative RT-PCR. The expression of miR-132 was increased in a time-dependent manner for 12 h when THP-1 cells were exposed to 200 μM PA (Figure 2A). Similarly, PA up-regulated miR-132 expression in primary murine macrophages (Figure 2B).

### 3.3. miR-132 Regulates PA Induced-NLRP3 Inflammasome Activation in THP-1 Cells and Primary Murine Macrophages

**Figure 3:**
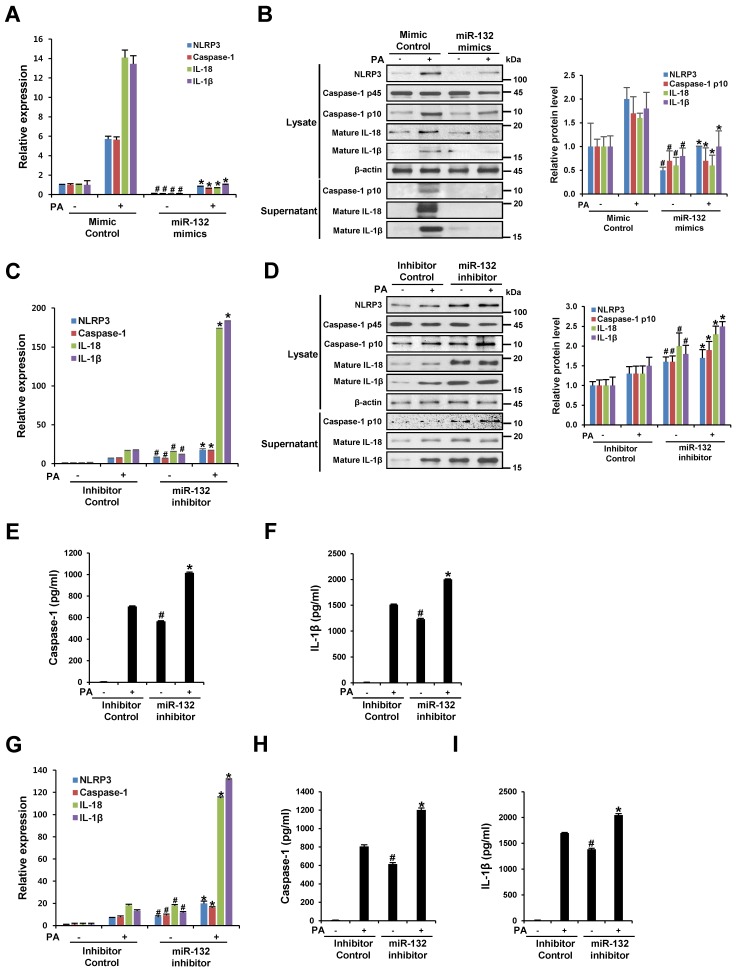
miR-132 regulates PA-induced NLRP3 inflammasome activation. (**A**–**F**) THP-1 cells were transfected with a mimic negative control, mimic, inhibitor negative control, or inhibitor of miR-132 at 20 nM for 48 h before being treated with 200 μM PA for 12 h; (**A**,**C**) The mRNA levels of NLRP3, caspase-1, IL-18, and IL-1β were examined by quantitative RT-PCR; (**B**,**D**) Cell lysates and supernatants were subjected to Western blot analysis using antibodies specific for NLRP3, caspase-1, IL-18, IL-1β, and β-actin. β-Actin was used as a loading control; (**E**,**F**) The levels of caspase-1 and IL-1β in the culture supernatants were determined by ELISA; (**G**–**I**) Primary murine macrophages were transfected with inhibitor negative control or inhibitor of miR-132 at 20 nM for 48 h before being treated with 200 μM PA for 12 h; (**G**) The mRNA levels of NLRP3, caspase-1, IL-18, and IL-1β were determined using quantitative RT-PCR; (**H**,**I**) The levels of caspase-1 and IL-1β in the culture supernatants were determined by ELISA. # *p* < 0.05 compared with the mimic or inhibitor negative control in PA-untreated cells, and * *p* < 0.05 compared with the mimic or inhibitor negative control in PA-treated cells.

To understand the role of miR-132 in NLRP3 inflammasome activation, THP-1 cells were transfected with a mimic negative control, miR-132 mimic, an inhibitor negative control, or miR-132 inhibitor, followed by stimulation with 200 μM PA for 12 h. Transfection of PA-untreated THP-1 cells with miR-132 mimic the significantly decreased mRNA expression of NLRP3, caspase-1, IL-18, and IL-1β compared to the mimic negative control (Figure 3A). In addition, the miR-132 mimic significantly down-regulated the increased mRNA levels of NLRP3, caspase-1, IL-18, and IL-1β induced by PA treatment compared to the mimic negative control (Figure 3A). Western blotting analysis of NLRP3 protein in cell lysates and supernatants revealed that the miR-132 mimic decreased the expression and release of the PA-induced NLRP3 inflammasome component (Figure 3B). In contrast, as shown in Figure 3C–F, the miR-132 inhibitor enhanced the mRNA and protein expression of the NLRP3 inflammasome component, and increased caspase-1 activity and IL-1β secretion compared to the inhibitor negative control in THP-1 cells. Furthermore, inhibition of miR-132 increased PA-induced NLRP3 inflammasome activation in primary murine macrophages (Figure 3G–I). These results imply that miR-132 is involved in PA-induced inflammasome activation, which can lead to caspase-1 activation as well as IL-1β and IL-18 processing and release.

### 3.4. miR-132 Does Not Regulate PA-Induced TLR Signaling in THP-1 Cells

**Figure 4:**
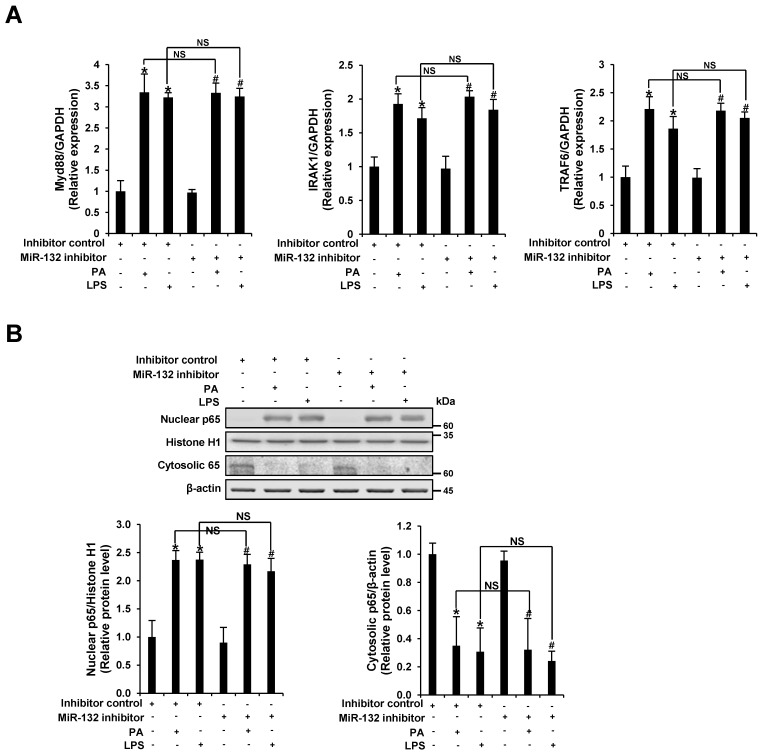
miR-132 does not regulate PA-induced TLR signaling. THP-1 cells were transfected with an inhibitor negative control or inhibitor of miR-132 at 20 nM for 48 h before being treated with 200 μM PA or 1 μg/mL LPS (as a positive control) for 12 h. (**A**) The mRNA levels of MyD88, IRAK1, and TRAF6 were examined by quantitative RT-PCR; (**B**) Nuclear and cytosolic fractions were subjected to Western blot analysis using antibodies specific for NF-κB p65, histone H1, and β-actin. Histone H1 and β-actin were used as a loading control. All results are representative of three independent experiments. * *p* < 0.05 compared with the inhibitor negative control-transfected cells, and # *p* < 0.05 compared with the miR-132 inhibitor-transfected cells. NS, no significance.

To determine whether miR-132 regulates PA-induced TLR signaling, THP-1 cells were transfected with an inhibitor negative control or miR-132 inhibitor, followed by stimulation with 200 μM PA for 12 h. The mRNA levels of myeloid differentiation primary response 88 (MyD88), IL-1 receptor-associated kinase 1 (IRAK1), and TNF receptor-associated factor 6 (TRAF6) were examined. PA up-regulated the mRNA expression of MyD88, IRAK1, and TRAF6, and no significant differences in PA-induced MyD88, IRAK1, and TRAF6 mRNA expression were detected between inhibitor negative control and miR-132 inhibitor transfected cells (Figure 4A). In addition, no significant difference in PA-induced NF-κB p65 protein expression was observed between inhibitor negative control and miR-132 inhibitor transfected cells (Figure 4B). These data suggest that miR-132 does not regulate PA-induced TLR signaling in THP-1 cells.

### 3.5. FOXO3 Is a Target of miR-132 

**Figure 5:**
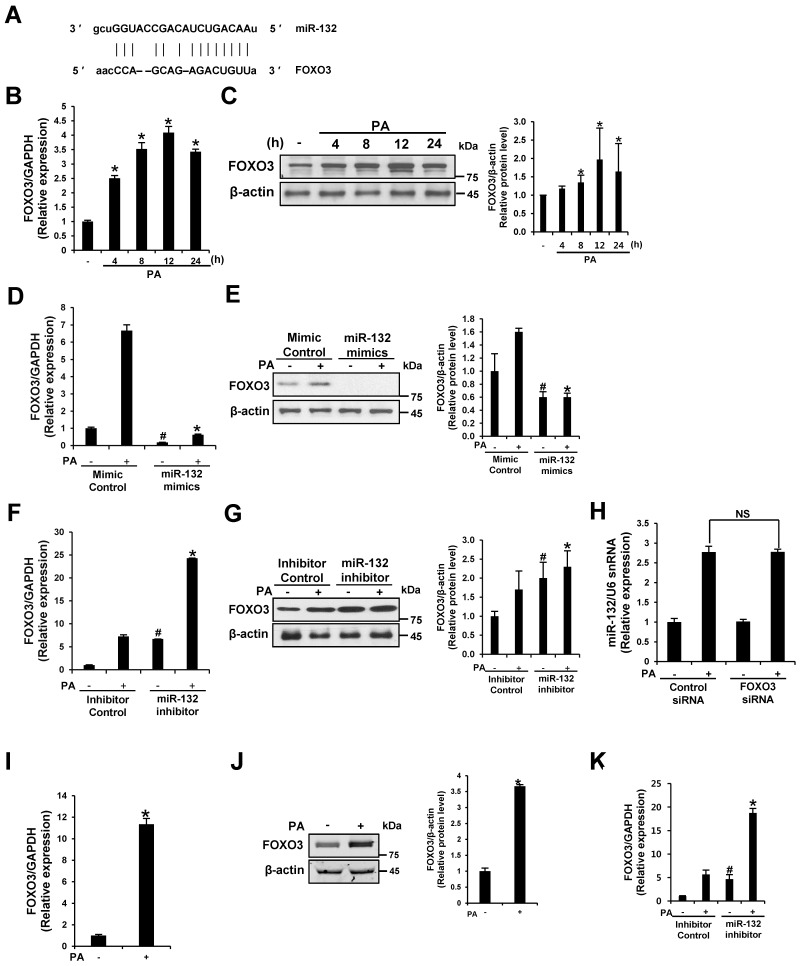
FOXO3 is a target of miR-132. (**A**) miR-132 targets were predicted using TargetScan (www.targetscan.org) and microRNA (www.microrna.org); (**B**) PA induces the mRNA expression of FOXO3; (**C**) PA induces the protein expression of FOXO3; (**D**–**G**) THP-1 cells were transfected with a mimic negative control, mimic, inhibitor negative control, or inhibitor of miR-132 at 20 nM for 48 h before treatment with 200 μM PA for 12 h; (**D**,**F**) The effect of the miR-132 mimic and inhibitor on the FOXO3 mRNA content was determined by quantitative RT-PCR; (**E**,**G**) Cell lysates were subjected to Western blot analysis using antibodies specific for FOXO3 and β-actin. β-Actin was used as a loading control; (**H**) The effect of FOXO3 silencing on miR-132 expression was determined by quantitative RT-PCR in THP-1 cells. NS, no significance; (**I**,**J**) Primary murine macrophages were treated with 200 μM PA for 12 h. Levels of FOXO3 mRNA and protein expression were measured; (**K**) Primary murine macrophages were transfected with inhibitor negative control or inhibitor of miR-132 at 20 nM for 48 h before being treated with 200 μM PA for 12 h. FOXO3 mRNA was examined by quantitative RT-PCR. # *p* < 0.05 compared with the mimic or inhibitor negative control in PA-untreated cells, and * *p* < 0.05 compared with the mimic or inhibitor negative control in PA-treated cells.

To elucidate the molecular functions of miR-132 in the regulation of PA-induced inflammasome activation, we screened predicted targets of these miRNAs in silico to identify genes related to inflammasome activation using TargetScan (www.targetscan.org) and microRNA (www.microrna.org); we identified FOXO3 (Figure 5A). FOXO3 was recently validated as a direct target of miR-132 in neurons and bone marrow cells [18,19]. PA up-regulated FOXO3 mRNA and protein expression in THP-1 cells and primary murine macrophages (Figure 5B,C,I,J). To investigate whether PA-induced FOXO3 activation is due to TLR-induced NF-κB activity, the mRNA levels of FOXO3 were examined in NF-κB-silenced cells. No significant difference in FOXO3 expression was detected between the siRNA control and NF-κB-silenced cells (Supplementary Materials Figure S1G).

To verify the miR-132 target FOXO3, we transfected a miR-132 mimic or miR-132 inhibitor into THP-1 cells and treated the cells with PA. The miR-132 mimic down-regulated FOXO3 mRNA and protein expression compared to the mimic negative control with or without PA treatment, while the miR-132 inhibitor up-regulated FOXO3 (Figure 5D–G,K). No significant difference in PA-induced miR-132 expression was observed between the siRNA control and FOXO3 siRNA-transfected cells (Figure 5H). Therefore, data from previous studies and our findings indicate that FOXO3 is a target of miR-132 in THP-1 cells.

### 3.6. The Silencing of FOXO3 Suppresses PA-Induced NLRP3 Inflammasome Activation in THP-1 Cells and Primary Murine Macrophages

**Figure 6:**
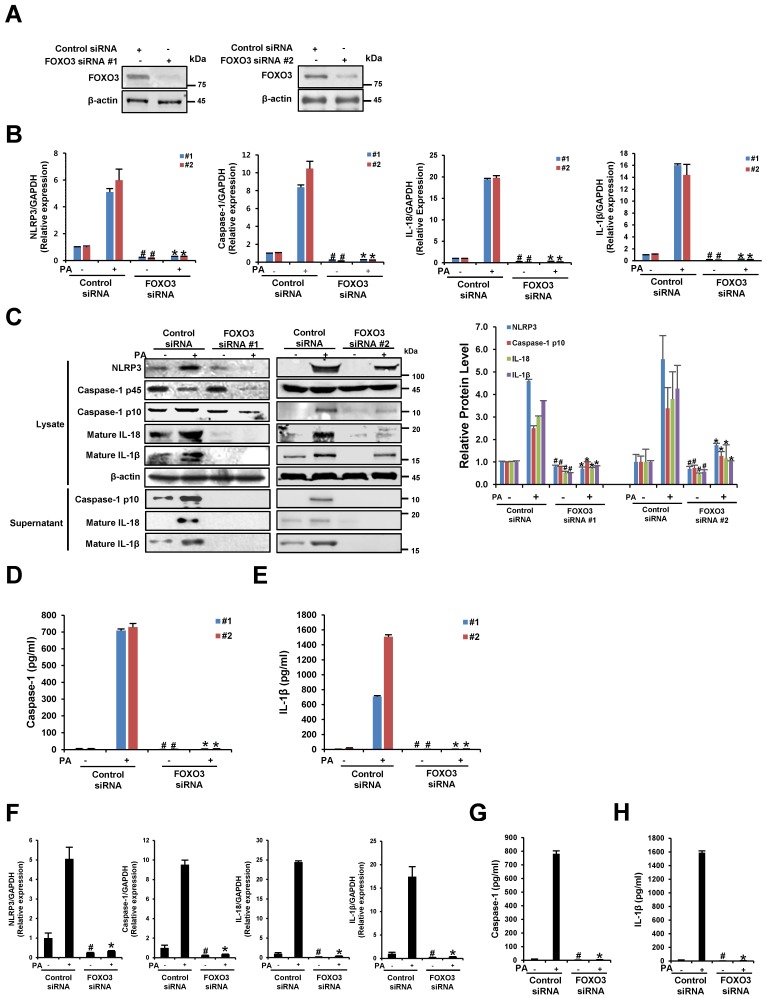
The silencing of FOXO3 suppresses PA-induced NLRP3 inflammasome activation. (**A**–**E**) THP-1 cells were transfected with 20 nM control siRNA or FOXO3 siRNA for 48 h and then treated with PA (200 μM) for 12 h; (**A**) The FOXO3 siRNA transfection efficiency was measured by Western blot analysis; (**B**) The mRNA levels of NLRP3, caspase-1, IL-18, and IL-1β were examined by quantitative RT-PCR; (**C**) Cell lysates and supernatants were subjected to Western blot analysis using antibodies specific for NLRP3, caspase-1, IL-18, IL-1β, and β-actin. β-Actin was used as a loading control; (**D**,**E**) The levels of caspase-1 and IL-1β in the culture supernatants were determined by ELISA; (**F**–**H**) Primary murine macrophages were transfected with 20 nM control siRNA or FOXO3 siRNA for 48 h and then treated with PA (200 μM) for 12 h; (**F**) The mRNA levels of NLRP3, caspase-1, IL-18, and IL-1β were examined by quantitative RT-PCR; (**G**,**H**) The levels of caspase-1 and IL-1β in the culture supernatants were determined by ELISA. # *p* < 0.05 compared with the control siRNA in PA-untreated cells, and * *p* < 0.05 compared with the control siRNA in PA-treated cells.

To understand the direct influence of FOXO3 on PA-induced NLRP3 inflammasome activation, the mRNA and protein levels of NLRP3, caspase-1, IL-18, and IL-1β were examined in FOXO3-silenced cells. The transfection efficiency in 48 h post-transfected cells was confirmed by Western blot analysis (Figure 6A). Compared to the scrambled siRNA control, FOXO3 siRNA-transfected cells exhibited a significant decrease in NLRP3, caspase-1, IL-18, and IL-1β gene expression in response to PA (Figure 6B,F). In addition, Figure 6C shows that the protein levels of NLRP3, caspase-1, IL-18, and IL-1β in lysates and supernatants prepared from FOXO3-silenced cells decreased with or without PA treatment. The silencing of FOXO3 down-regulated PA-induced caspase-1 activity and IL-1β secretion (Figure 6D,E,G,H). These results suggest that the suppression of FOXO3 inhibits the activation of NLRP3 inflammation.

### 3.7. NLRP3 Inflammasome Activation through miR-132 Inhibitor Is Attenuated by the Knockdown of FOXO3

**Figure 7:**
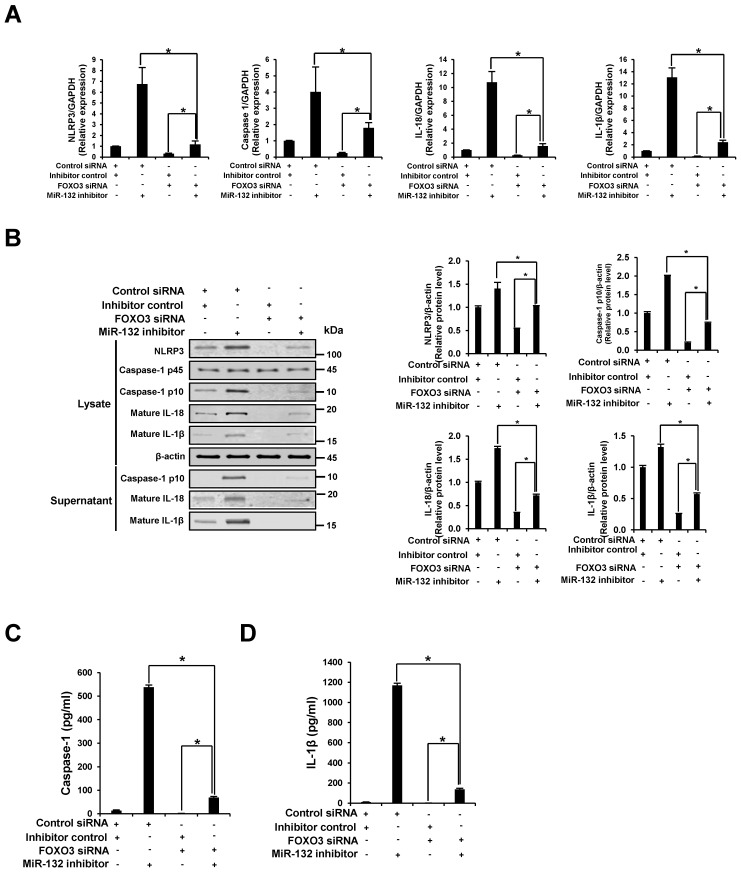
NLRP3 inflammasome activation through miR-132 inhibitor is attenuated by the knockdown of FOXO3. THP-1 cells were co-transfected with 20 nM inhibitor negative control or miR-132 inhibitor plus 20 nM control or FOXO3 siRNA for 48 h. (**A**) The mRNA levels of NLRP3, caspase-1, IL-18, and IL-1β were examined by quantitative RT-PCR; (**B**) Cell lysates and supernatants were subjected to Western blot analysis using antibodies specific for NLRP3, caspase-1, IL-18, IL-1β, and β-actin. β-Actin was used as a loading control; (**C**,**D**) The levels of caspase-1 and IL-1β in the culture supernatants were determined by ELISA. All results are representative of three independent experiments. * *p* < 0.05 between the two groups.

We examined whether the knockdown of FOXO3 counteracts the NLRP3 inflammasome activation effect by inhibition of miR-132. Co-transfection with FOXO3 siRNA, but not control siRNA, significantly diminished the increase in mRNA and protein expression of the NLRP3 inflammasome component by miR132 inhibitor (Figure 7A,B). A similar pattern was observed in cells co-transfected with FOXO3 siRNA, showing decreased miR-132 inhibitor-induced caspase-1 activation and secretion of IL-1β (Figure 7C,D). These findings indicate that miR-132 regulates NLRP3 inflammasome activation through down-regulation of FOXO3.

### 3.8. Schematic Diagram of the Proposed Model Showing How miR-132 Regulates PA-Induced NLRP3 Inflammasome Activation 

**Figure 8:**
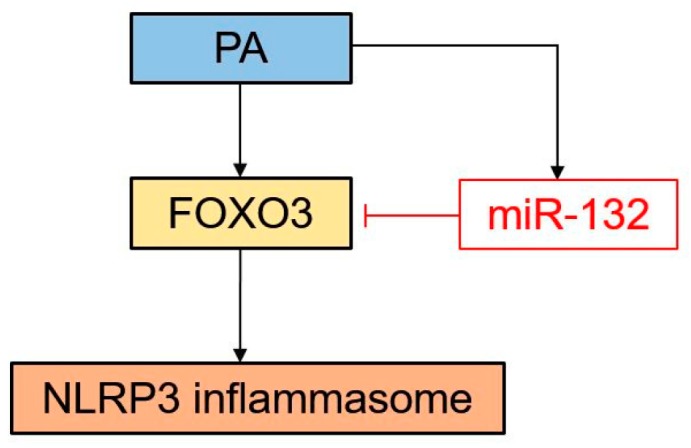
Schematic diagram of the proposed model showing the role of miR-132 in PA-induced NLRP3 inflammasome activation. PA up-regulates activation of the NLRP3 inflammasome, miR-132, and FOXO3. miR-132 negatively regulates NLRP3 inflammasome activation through FOXO3 down-regulation.

On the basis of our findings, we suggest the model below (Figure 8). PA induces the activation of the NLRP3 inflammasome and up-regulates FOXO3 and miR-132 expression. FOXO3, as a target of miR-132, is involved in PA-induced NLRP3 inflammasome activation. miR-132 serves as a negative feedback regulator in PA-induced NLRP3 inflammasome activation via FOXO3 down-regulation.

## 4. Discussion

Here, we report a potential mechanism of miR-132 in the regulation of PA-triggered NLRP3 inflammasome activation in THP cells and primary murine macrophages. Our results show that PA induced miR-132 expression as well as NLRP3 inflammasome activation. Overexpression of miR-132 was associated with a significant decrease in FOXO3 protein expression, along with a reduction in NLRP3, caspase-1 p10, IL-18, and IL-1β production in response to PA stimulation. Suppression of miR-132 resulted in the opposite results. In addition, silencing of FOXO3 suppressed PA-induced NLRP3 inflammasome activation. NLRP3 inflammasome activation through miR-132 inhibitor is attenuated by the knockdown of FOXO3. Collectively, these data suggest that miR-132 is an important modulator that plays a negative role in PA-induced NLRP3 inflammasome activation, at least in part through FOXO3.

Emerging evidence suggests that NLRP3 activation is involved in inflammatory responses, antimicrobial responses, and various human diseases, including autoimmune inflammatory disease, Alzheimer’s disease, atherosclerosis, and type 2 diabetes [20]. Previous studies using genetically modified mice lacking NLRP3 inflammasome components showed that activation of the NLRP3 inflammasome is essential for the induction of obesity-induced inflammation and development of insulin resistance [9,10,21]. PA, one of the most abundant SFAs in the modern diet induces activation of the NLRP3 inflammasome in hematopoietic cells, which results in insulin resistance and glucose intolerance in mice [10]. In contrast, unsaturated fatty acids show a preventive effect on the activation of the NLRP3 inflammasome [22]. Furthermore, NLRP3 inflammasome activation is elevated in myeloid cells from type 2 diabetic patients [23]. 

The regulation of NLRP3 inflammasome-mediated IL-1β production in mononuclear phagocytes is cell-type specific [24]. In this study, untreated THP-1 cells expressed NLRP3 protein and released active caspase-1, IL-18, and IL-1β into the supernatant, and this was enhanced by PA treatment. These results suggest that the NLRP3 inflammasome is constitutively active in THP-1 cells. Therefore, primary signaling that induces the expression of pro-IL-1β mediated by the activation of TLRs through PA is sufficient for the production of IL-1β in THP-1 cells [22]. PA also potentiates the inflammasome by up-regulating the expression of NLRP3. 

We found that miR-132 regulated PA-induced NLRP3 inflammasome activation, but not TLR signaling. Modulation of miR-132 had no effect on PA-induced MyD88, IRAK1, TRAF6, and NF-κB p65 expression, which are known to be important in TLR-4 and pro-inflammatory signaling. Therefore, the regulatory effect of miR-132 on NLRP3 inflammasome activation may not be associated with TLR signaling as an upstream pathway. 

As for FOXO3, the silencing of NF-κB had no effect on PA-induced FOXO3 expression. Although LPS can induce NLRP3 inflammasome activation and miR-132, it did not induce FOXO3 in THP-1 cells (Supplementary Figure S1A–F). These results suggest that PA-induced FOXO3 activation may not be the downstream effect of TLR-induced NF-κB activity. In addition, recent studies have demonstrated non-canonical activation of the inflammasome by LPS via caspase-4 and caspase-5, independent of the traditional LPS receptor TLR4 [25]. Thus, this may lead to differences in FOXO3 expression induced by PA and LPS. Further study is needed to clarify these differences. 

The mechanism by which FOXO3 activates the NLRP3 inflammasome remains unknown. It has been reported that uncoupling protein 2 (UCP2), a known FOXO3 target, promotes NLRP3 inflammasome activation in macrophages [26]. Therefore, in an indirect pathway, it is possible that FOXO3 activates the NLRP3 inflammasome through UCP2. Additional studies on the direct action of FOXO3 on the NLRP3 inflammasome are necessary to confirm this indirect hypothesis.

Several studies have demonstrated the function of miR-132. miR-132 has also been implicated in neuronal differentiation and function [27,28]. miR-132 attenuates neuroinflammation by targeting acetylcholinesterase, and it can modulate inflammation induced by bacterial infection [14,29]. miR-132, which is induced by LPS or peptidoglycan, can act via negative feedback to prevent uncontrolled inflammation. In addition, miR-132 can regulate Kaposi’s sarcoma-associated herpesvirus-induced inflammation [15]. Overexpression of miR-132 has been shown in chronic autoimmune disease conditions [30,31] and hematological malignancies [32]. Taken together, this suggests that miR-132 is an important regulator of the immune system. 

To our knowledge, our study is the first to show the role of miR-132 in PA-induced NLRP3 inflammasome activation. In the current study, the mRNA expression levels of the NLRP3 inflammasome components, miR-132 expression, and FOXO3 mRNA expression were concurrently increased approximately 4 h after PA treatment. The protein expression levels of FOXO3 and the NLRP3 inflammasome components were increased 8 and 12 h after PA treatment, respectively. Furthermore, NLRP3 inflammasome activation through miR-132 inhibition was attenuated by FOXO3 knockdown. These findings suggest that miR-132 acts as a tuning regulator to prevent overactivation of the inflammasome by downregulating FOXO3.

Based on studies by Park et al. [33,34], even though *Porphyromonas gingivalis* induced IL-1β release via NLRP3 inflammasome activation and miR-132 expression, miR-132 did not affect inflammasome activation in THP-1 cells. The precise reasons for this difference are unclear, but it may be due to the different stimuli used. 

The observed miR-132-mediated suppression of FOXO3 is similar to the findings of previous studies [18,19]. Until now, the relationship between miR-132 and FOXO3 has mostly been investigated in the context of cell survival because FOXO3 is a known aging-related pro-apoptotic transcription factor. Our results indicate that miR-132 suppresses inflammation activation through FOXO3.

## 5. Conclusions

In summary, our study identifies miR-132 as an important and novel negative feedback regulator of PA-induced NLRP3 inflammasome activation in THP-1 cells. Our results suggest that modulating miR-132 levels could be used in therapeutic interventions for inflammasome activation stimulated by high fat.

## Figures and Tables

**Table 1:** Primer sequences used for quantitative RT-PCR.

	Gene	Sequence 5′ to 3′
Human	NLRP3	F: ACAGCCACCTCACTTCCAG, R: CCAACCACAATCTCCGAATG
	Caspase-1	F: GCACAAGACCTCTGACAGCA, R: TTGGGCAGTTCTTGGTATTC
	IL-18	F: GCCTAGAGGTATGGCTGTAA, R: GCGTCACTACACTCAGCTAA
	IL-1β	F: GCCCTAAACAGATGAAGTGCTC, R: GAACCAGCATCTTCCTCAG
	FOXO3	F: CGGACAAACGGCTCACTCT, R: GGACCCGCATGAATCGACTAT
	MyD88	F: GAGCGTTTCGATGCTTCAT, R: CGGATCATCTCCTGCACAAA
	IRAK1	F: ACTGGCCCTTGGCAGCTC, R: GGCCAGCTTCTGGACCATC
	TRAF6	F: CCTTTGGCAAATGTCATCTGTG, R: CTCTGCATCTTTTCATGGCAAC
	GAPDH	F: AAAATCAAGTGGGGCGATGC, R: AGGAGGCATTGCTGATGATCT
Mouse	NLRP3	F: CGAGACCTCTGGGAAAAAGCT, R: GCATACCATAGAGGAATGTGATGTACA
	Caspase-1	F: GATGGCACATTTCCAGGACTGA, R: TGTTGCAGATAATGAGGGCAAGAC
	IL-18	F: GTGAACCCCAGACCAGACTG, R: CCTGGAACACGTTTCTGAAAGA
	IL-1β	F: GAAATGCCACCTTTTGACAGTG, R: TGGATGCTCTCATCAGGACAG
	FOXO3	F: AGCCGTGTACTGTGGAGCTT, R: TCTTGGCGGTATATGGGAAG
	GAPDH	F: TGACCTCAACTACATGGTCTACA, R: CTTCCCATTCTCGGCCTTG

## Supplementary Materials

The Supplementary file is available online at www.mdpi.com/2072-6643/9/12/1370/s1.
