# Supplementary material for: MicroRNA-132 Negatively Regulates Palmitate-Induced NLRP3 Inflammasome Activation through FOXO3 Down-Regulation in THP-1 Cells
Source: Nutrients. 2017 Dec 18;9(12):1370. doi: 10.3390/nu9121370 (PMC5748820; doi:10.3390/nu9121370)
Supplement: Supplementary file 1 [file nutrients-09-01370-s001.pdf]

Supplementary Figure S1. THP-1 cells were treated with 200 μM PA or 1 μg/mL LPS (as a positive control) for 12 h. (**A**) The mRNA levels of NLRP3, caspase-1, IL-18, and IL-1β were examined by quantitative RT-PCR. (**B**) Cell lysates and supernatants were subjected to immunoblotting using antibodies specific for NLRP3, caspase-1, IL-18, IL-1β, and β-actin. Relative protein levels were analyzed with ImageJ. (**C**) The levels of caspase-1 and IL-1β in the culture supernatants were determined by ELISA. *$p < 0.05$ compared with PA-untreated THP-1 cells. (**D**) miR-132 expression was analyzed by quantitative RT-PCR. *$p < 0.05$ compared with PA-untreated THP-1 cells. (**E**,**F**) FOXO3 mRNA and protein expression were analyzed by quantitative RT-PCR and Western blotting. *$p < 0.05$ compared with PA-untreated THP-1 cells. (**G**) The effect of NF-κB silencing on PA-induced FOXO3 expression was determined by quantitative RT-PCR in THP-1 cells. NS, no significance.

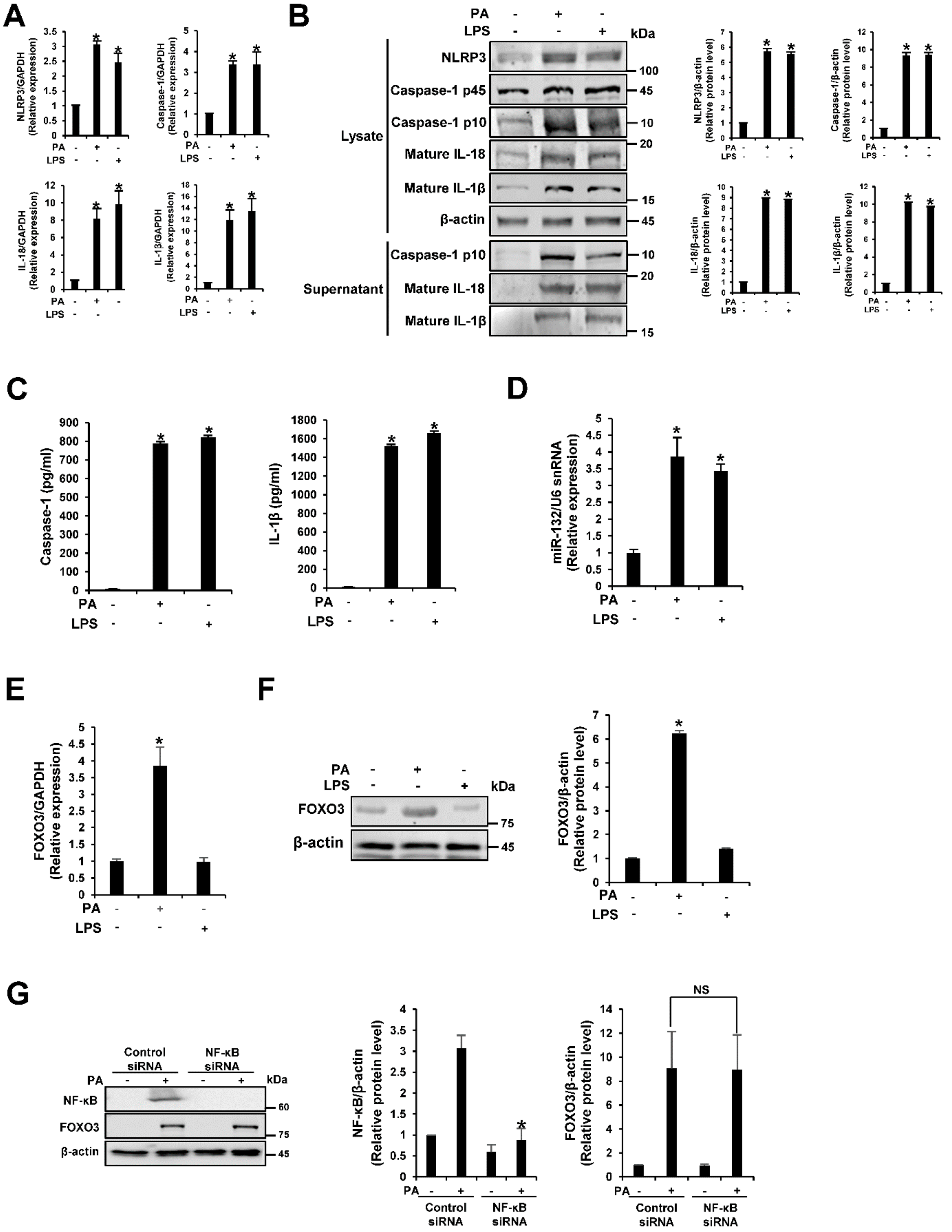
A
NLRP3/GAPDH (Relative expression)
Caspase-1/GAPDH (Relative expression)
IL-18/GAPDH (Relative expression)
IL-1β/GAPDH (Relative expression)
PA
LPS
B
PA
LPS
kDa
Lysate
NLRP3
Caspase-1 p45
Caspase-1 p10
Mature IL-18
Mature IL-1β
β-actin
Supernatant
NLRP3/β-actin (Relative protein level)
Caspase-1/β-actin (Relative protein level)
IL-18/β-actin (Relative protein level)
IL-1β/β-actin (Relative protein level)
C
Caspase-1 (pg/ml)
IL-1β (pg/ml)
D
miR-132/U6 snRNA (Relative expression)
E
FOXO3/GAPDH (Relative expression)
F
FOXO3
FOXO3/β-actin (Relative protein level)
G
Control siRNA
NF-κB siRNA
NF-κB
NF-κB/β-actin (Relative protein level)
NS
